# A comparison of clinical outcomes and optical performance between monofocal and new monofocal with enhanced intermediate function intraocular lenses: a case-control study

**DOI:** 10.1186/s12886-021-02124-w

**Published:** 2021-10-16

**Authors:** Jungah Huh, Youngsub Eom, Seul Ki Yang, Young Choi, Hyo Myung Kim, Jong Suk Song

**Affiliations:** 1 Department of Ophthalmology, Korea University College of Medicine, Seoul, Republic of Korea; 2 Department of Ophthalmology, Korea University Guro Hospital, 148 Gurodong-ro, Guro-gu, Seoul, 08308 South Korea; 3 Department of Ophthalmology, Korea University Ansan Hospital, Ansan-si, Gyeonggi-do Republic of Korea; 4 Space Optics Laboratory, Department of Astronomy, Yonsei University, Seoul, Republic of Korea; 5 Satellite system 2 Team, Hanwha Systems Co., Ltd., Yongin-si, Gyeonggi-do Republic of Korea

**Keywords:** Monofocal, Eyhance, Optical bench

## Abstract

**Background:**

To compare clinical outcomes and optical performance of a new monofocal with enhanced intermediate function intraocular lenses (IOLs) with that of conventional monofocal IOLs.

**Methods:**

Sixty eyes of 30 patients who underwent phacoemulsification with bilateral implantation of the ICB00 (15 patients) or ZCB00 (15 patients) IOLs were enrolled. Binocular corrected distance visual acuity (CDVA), distance corrected near visual acuity (DCNVA), and distance corrected intermediate visual acuity (DCIVA) were measured at 4 weeks after surgery. Patient satisfaction for overall, near, intermediate, and distance vision were assessed. The binocular defocus curves were measured. The root mean square of modulation transfer function (MTF_RMS_) was measured in the optical bench study.

**Results:**

The mean binocular DCIVA was significantly better in the ICB00 group (0.01 logMAR) compared to the ZCB00 group (0.13 logMAR), but CDVA and DCNVA were not. The patient satisfaction for near and intermediate vision was significantly higher in the ICB00 group compared to the ZCB00. However, there was no difference in patient satisfaction for overall and distance vision between two groups. The defocus curves showed that mean visual acuity of the ICB00 group was significantly better than that of the ZCB00 group at between − 1.00 D to − 3.00 D of defocus. The ICB00 IOL had higher MTF_RMS_ values at between − 0.50 D to − 2.00 D of defocus compared to the ZCB00 IOL.

**Conclusions:**

The ICB00 IOL provides better binocular intermediate vision and higher satisfaction for near and intermediate vision than the ZCB00 IOL while maintaining excellent distance vision.

## Background

Cataracts are the most common cause of impaired vision worldwide, and cataract surgery is the most common surgical procedure in the field of ophthalmology [1, 2]. Cataract surgery with conventional monofocal intraocular lens (IOL) implantation has shown very successful results for distant vision, but patients often require spectacle correction for near vision [3]. Multifocal IOLs have been developed to meet patients’ need for near vision, but there is a limit to the increased incidence of subjective visual disturbance, including halos and glare [4–6].

Currently, extended computer use and younger age at cataract surgery also give rise to growing needs for intermediate vision [7–9]. The newly developed TECNIS Eyhance ICB00 (Johnson & Johnson Vision Care, Inc.), a monofocal with a higher-order aspheric anterior surface IOL to enhance intermediate function, sought to meet those needs while sparing distant vision and visual disturbance. It shares the same geometry with the conventional monofocal TECNIS 1-piece ZCB00 IOL (Johnson & Johnson Vision Care, Inc.) about 85% of the surface except for the modified aspherical anterior surface of the optics [10]. This unique anterior surface is intended to create a continuous power change from the periphery to the center inducing the continuous power profile created with a higher-order asphere and improves intermediate vision. It is based on the refractive technology, without diffractive rings or zones, and it is visually indistinguishable from the TENIS 1-piece ZCB00 IOL. Thus, we wanted to know how the visual performance improved as the anterior surface profile changed from the ZCB00 to ICB00.

The purpose of this study was to compare clinical outcomes in terms of distance, near, and intermediate visual acuities, visual disturbances, and spectacle independence between patients who underwent bilateral implantation of ZCB00 IOLs and patients who underwent bilateral implantation of ICB00 IOLs. Besides, we also evaluated the optical performance of two IOLs through optical bench testing.

## Methods

### Study population

This retrospective case-control study included patients who underwent cataract surgery with either the ZCB00 or ICB00 IOLs implanted bilaterally at the Korea University College of Medicine between March and October 2020. Patient who had a postoperative corrected distance visual acuity (CDVA) of 20/40 or better in the operated eye were included. Eyes with traumatic cataracts, a previous history of ocular surgery, eventful surgery (eg, anterior capsule tear), or postoperative complications were excluded. This study adhered to the tenets of the Declaration of Helsinki, and was approved by both the Institutional Review Board of Korea University Guro Hospital (IRB no. 2020GR0525) and that of Korea University Ansan Hospital (IRB no. 2020AS0344). The Institutional Review Board of Korea University Medicine waived the need for written informed consent from the participants, because of the study’s retrospective design.

### Patient examination

Preoperative uncorrected distance visual acuity (UDVA) was measured at 4 m. The preoperative corneal power, anterior chamber depth (ACD), and axial length (AL) were measured using an IOLMaster 500 (Carl Zeiss Meditec AG, Jena, Germany). The IOL power was calculated based on the predicted refraction by Haigis formula, and targeted between 0 and − 0.50 D. IOL constants of a0, a1, and a2 for the Haigis formula were − 1.302, 0.210, and 0.251, respectively.

### Surgical technique

All phacoemulsification and IOL implantations were performed by one of two experienced surgeons (S.J.S. and E.Y.) in one of our two institutions under topical anesthesia with 0.5% proparacaine hydrochloride (Alcaine; Alcon Laboratories Inc., Fort Worth, Tx or Paracaine; Hanmi Pharm, Seoul, Korea). After making a 2.20- or 2.75-mm clear corneal incision, a 26-gauge needle and a capsulorrhexis forceps were used to create a continuous curvilinear capsulorrhexis slightly smaller than the IOL optic size. The phacoemulsification was performed with either the stop-and-chop or phaco-chop technique. The IOL was folded for implantation using an insertion system, and inserted into the capsular bag through a clear corneal incision. All patient was treated with topical 1.5% levofloxacin (Cravit; Santen Pharmaceutical, Osaka, Japan) and topical steroid eyedrop (1% prednisolone acetate (Pred Forte®; Allergan, Inc., Irvine, CA) or 0.1% fluorometholone (Santen Pharmaceutical)) 4 times daily, and 0.1% bromfenac sodium (Bronuck; Taejoon Pharm, Seoul, Korea) twice daily from 3 days before cataract surgery to 4 weeks after cataract surgery.

### Patient evaluation

Postoperative monocular and binocular uncorrected and corrected distance visual acuity (CDVA) at 4 m, binocular uncorrected and distance corrected near visual acuity (UNVA, DCNVA) at 40 cm, and binocular uncorrected and distance corrected intermediate visual acuity (UIVA, DCIVA) at 66 cm were measured at postoperative visits 4 weeks after surgery. The distance corrected defocus curves were obtained binocularly at 4 m to measure the visual acuity with each defocus between − 3.00 D and + 1.00 D in 0.50 D intervals [6].

The refractive prediction error was defined as the difference between postoperative achieved refraction and preoperative targeted refraction (i.e., refractive prediction error = achieved spherical equivalent – targeted spherical equivalent). Mean absolute error (MAE) was defined as the mean absolute value of refractive prediction error and median absolute error (MedAE) was defined as the median absolute value of refractive prediction error [11].

Postoperative incidence of photic phenomena (glare, starburst, and halos), patient satisfaction for overall, near, intermediate, and distance vision, and dependence on glasses for near, intermediate, and distance vision were assessed with a questionnaire answered at 4 weeks after cataract surgery [6, 12]. A questionnaire with illustrations was used to assess whether patients experienced photic phenomena after cataract surgery. Patient satisfaction was rated on a 1-5 scale: 1, Very dissatisfied; 2, Dissatisfied; 3, Neutral; 4, Satisfied; 5, Very satisfied. Spectacle dependence was rated on a 1-5 scale: 1, Never; 2, Seldom; 3, About half the time; 4, Usually; 5, Always (Table 1) [12].

**Table 1:** Questionnaire used for comparison between monofocal and new monofocal with enhanced intermediate function intraocular lenses

Questions	Answer
1. Do you experience glare?	Yes / No
2. Do you experience starbursts?	Yes / No
3. Do you experience halos?	Yes / No
4. How do you satisfied with your overall visual acuity?	1-5 scale^a^
5. Do you need glasses for near vision (book)?	1-5 scale^b^
6. How do you satisfied with your near visual acuity?	1-5 scale^a^
7. Do you need glasses for intermediate vision (computer)?	1-5 scale^b^
8. How do you satisfied with your intermediate visual acuity?	1-5 scale^a^
9. Do you need glasses for distance vision (TV)?	1-5 scale^b^
10. How do you satisfied with your distance visual acuity?	1-5 scale^b^

^a^Patient satisfaction was rated on a 1-5 scale: 1, Very dissatisfied; 2, Dissatisfied; 3, Neutral; 4, Satisfied; 5, Very satisfied

^b^Spectacle dependence was rated on a 1-5 scale: 1, Never; 2, Seldom; 3, About half the time; 4, Usually; 5, Always

### Optical bench system

**Fig. 1:**
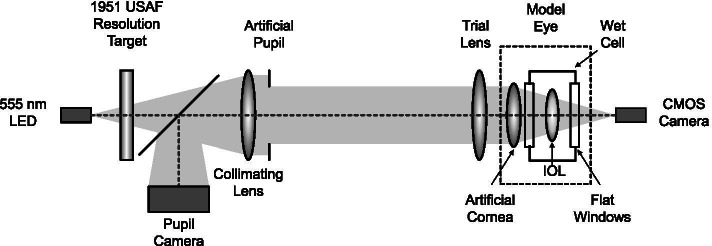
Schematic diagram of the optical bench system used in this study (LED = light emitting diode; USAF = United States Air Force; IOL = intraocular lens; CMOS = complementary metal–oxide–semiconductor)

The optical bench system used in this study consisted of a LED light, the 1951 United States Air Force (1951 USAF) resolution test chart, an artificial pupil, a pupil camera, trial lens, model eye, and complementary metal-oxide-semiconductor (CMOS) camera (BFS-U3-120S4M-CS; FLIR Systems Inc., Wilsonville, OR) (Fig. 1) [13]. The model eye composed of an aberration-free artificial cornea and a wet cell which was made of N-BK7 (DG100X100-600) and filled with a balanced salt solution, and they were mounted on the XYZ translation stage [14]. The IOL was fixed using an aspheric lens adapter which was mounted on the XYZ translation stage and then positioned in a wet cell. After that the lenses center and the camera were precisely aligned so that the image focus was on the camera’s sensor. The 1951 USAF resolution chart was illuminated by 555 nm LED light, and the image formed by the model eye was obtained by the CMOS camera [14, 15]. The trial lens was placed in front of the model eye to obtain defocus image between − 2.50 D and + 1.00 D in 0.50 D intervals [16]. These measurements were repeated at different pupil size from 2.0 mm to 5.0 mm in 1.0 mm increments using the artificial pupil with scale.

The images of the 1951 USAF target formed by two IOLs of 21.0 D ZCB00 and ICB00 IOLs were compared following International Organization for Standardization (ISO) 11,979-2 requirements and test methods (International Organization for Standardization, 2014. Ophthalmic Implants – Intraocular Lenses – Part 2: Optical Properties and Test Methods). The element 3 of the group 2 in the 1951 USAF resolution test chart was set to be 15 cycle per degree (CPD), which is approximately equivalent to 20/40 vision. The images were converted to through-focus modulation transfer function (MTF) in the horizontal and vertical directions using the MatLab (Mathworks, Inc., Natick, MA) [17]. The root mean square of the horizontal and vertical MTF (MTF_RMS_) was calculated using the following formula [15]:

$$MTF=\frac{I_{max}-{I}_{min}}{I_{max}+{I}_{min}}\kern0.5em {MTF}_{RMS}=\sqrt{\frac{MTF_{Horizontal}^2+{MTF}_{Vertical}^2}{2}}$$

### Statistical analysis

Data analysis was performed using the Statistical Package for the Social Sciences (version 20.0, SPSS, Inc.). Student’s t tests, Mann-Whitney U tests, and Fisher’s exact tests were performed to compare the clinical characteristics, IOLMaster 500 measurements, implanted IOL power, monocular UDVA, monocular and binocular CDVA, binocular CDVA, UNVA, DCNVA, UIVA, and DCIVA, incidence of photic phenomena, patient satisfaction score, and spectacle dependence score between the ZCB00 and ICB00 groups. A *P* value less than 0.05 was considered statistically significant.

## Results

### Clinical study

This study evaluated 60 eyes of 30 patients who underwent uncomplicated phacoemulsification with bilateral implantation of the TECNIS 1-piece ZCB00 (30 eyes of 15 patients) or TECNIS Eyhance ICB00 (30 eyes of 15 patients) IOL. The mean age of the 30 enrolled patients was 70.1 ± 7.1 years (range: 52–83 years). Among the total study population, there were 19 women (63.3%). There was no significant difference in mean age, sex ratio, preoperative UDVA, or mean corneal power between the two groups. On the other hand, the ZCB00 group showed a shallower ACD, shorter AL, and higher IOL power compared to the ICB00 group (Table 2).

**Table 2:** Baseline characteristics of patients with cataract and their eyes in a comparison between monofocal and new monofocal with enhanced intermediate function intraocular lenses

	ICB00 (30 eyes of 15 patients)	ZCB00 (30 eyes of 15 patients)	*P* value^a^
Age, y	69.6 ± 7.3	70.6 ± 7.2	0.708
Sex, Male: Female (%)	5 (33.3): 10 (66.7)	6 (40.0): 9 (60.0)	> 0.999^b^
UDVA, logMAR	0.37 ± 0.32	0.33 ± 0.34	0.591
Corneal power, D^c^	43.78 ± 1.31	44.26 ± 1.39	0.180
ACD, D^c^	3.32 ± 0.32	3.13 ± 0.25	0.015
AL, mm^c^	24.13 ± 1.38	23.36 ± 0.66	0.009
IOL power, D	19.9 ± 4.3	21.8 ± 1.3	0.027

Data are presented as mean ± standard deviation except sex

*UDVA* Uncorrected distance visual acuity, *logMAR* Logarithm of minimum angle of resolution, *D* Diopters, *ACD* Anterior chamber depth, *AL* Axial length, *IOL* Intraocular lens

^a^Student’s *t* test

^b^Chi-square test

^c^Corneal power, anterior chamber depth, and axial length measured by IOLMaster 500

Mean targeted and achieved refraction of the ICB00 group were − 0.23 ± 0.31 and − 0.16 ± 0.37 D, respectively, and those of the ZCB00 group were − 0.27 ± 0.19 and − 0.26 ± 0.34 D, respectively. There was no significant difference in MedAE and MAE between the ICB00 and ZCB00 groups (Table 3).

**Table 3:** Comparison of median absolute error and mean refractive prediction error calculated using the Haigis formula between the ICB00 and ZCB00 groups

	ICB00 (30 eyes of 15 patients)	ZCB00 (30 eyes of 15 patients)	*P* value^‡^
Targeted refraction, D^a^	−0.23 ± 0.31	−0.27 ± 0.19	0.628
Achieved refraction, D^a^	−0.16 ± 0.37	−0.26 ± 0.34	0.262
MedAE, D^b^	0.28 (0.18:0.45)	0.23 (0.13:0.44)	0.281^c^
MAE, D^a^	0.34 ± 0.23	0.28 ± 0.21	0.315

*D* Diopters, *MedAE* Median absolute error, *MAE* Mean absolute error

^‡^Student’s *t* test

^a^Values are presented as mean ± standard deviation

^b^Values are presented as median (interquartile range)

^c^Mann-Whitney U test

Table 4 shows the comparison of postoperative visual acuity between the ICB00 and ZCB00 groups. The mean monocular UDVA and CDVA, and binocular CDVA at 4 m was 0.06, − 0.01, and − 0.04 logMAR for the ICB00 group, respectively, 0.07, − 0.02, and − 0.05 logMAR for the ZCB00 group, respectively, with no significant differences between the two groups. On the other hand, the mean binocular UNVA was significantly better in the ICB00 group (0.09 logMAR) compared to the ZCB00 group (0.35 logMAR) (*p* <  0.001). Besides, the binocular UIVA and DCIVA were also better in the ICB00 group (0.03 and 0.01 logMAR) compared to the ZCB00 group (0.25 and 0.13 logMAR) (*p* <  0.001, *p* = 0.031, respectively).

**Table 4:** Comparison of postoperative visual acuity between the ICB00 and ZCB00 groups

	ICB00	ZCB00	*P* value*
Monocular UDVA at 4 m, logMAR	0.06 ± 0.10	0.07 ± 0.09	0.771
Monocular CDVA at 4 m, logMAR	− 0.01 ± 0.09	−0.02 ± 0.09	0.512
Binocular CDVA at 4 m, logMAR	− 0.04 ± 0.09	−0.05 ± 0.08	0.782
Binocular UNVA at 40 Cm, logMAR	0.09 ± 0.14	0.35 ± 0.14	< 0.001
Binocular DCNVA at 40 Cm, logMAR	0.09 ± 0.15	0.20 ± 0.17	0.075
Binocular UIVA at 66 Cm, logMAR	0.03 ± 0.06	0.25 ± 0.18	< 0.001
Binocular DCIVA at 66 Cm, logMAR	0.01 ± 0.04	0.13 ± 0.19	0.031

Values are presented as mean ± standard deviation

*UDVA* Uncorrected distance visual acuity, *CDVA* Corrected distance visual acuity, *UNVA* Uncorrected near visual acuity, *DCNVA* Distance corrected near visual acuity, *UIVA* Uncorrected intermediate visual acuity, *DCIVA* Distance corrected intermediate visual acuity, *logMAR* Logarithm of minimum angle of resolution

*Student’s *t* test

**Fig. 2:**
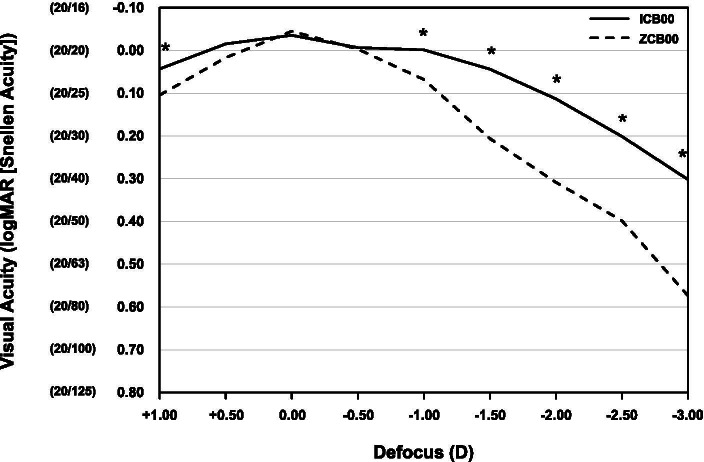
Mean binocular distance-corrected defocus curves at 4 weeks after cataract surgery of the ICB00 (solid line) and ZCB00 (dashed line) groups. An asterisk indicates *p* < 0.05 by the Student’s *t*-test

Figure 2 shows binocular distance-corrected defocus curves at 4 weeks after cataract surgery of the two groups. Both curves peaked at 0.00 D of defocus and decreased with increasing negative defocus. However, the ICB00 group achieved a smooth landing area with a less abrupt decrease in visual acuity, especially within the intermediate defocus range up to − 1.50 D. The mean visual acuity was 0.1 logMAR or more between + 1.00 D to − 1.50 D of defocus in the ICB00 group. The mean visual acuity of the ICB00 group was significantly better than that of the ZCB00 group at between − 1.00 D to − 3.00 D of defocus.

**Fig. 3:**
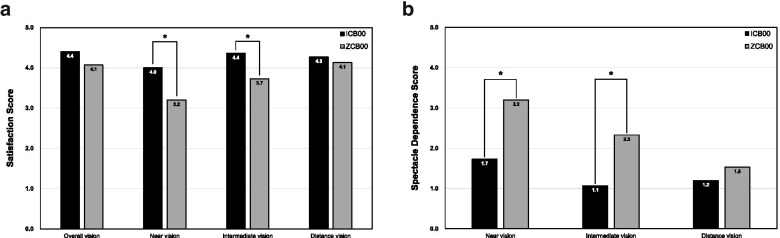
**a** Patient satisfaction score rated on a five-point scale as follows for overall, near, intermediate, and distance vision: 1, very dissatisfied; 2, dissatisfied; 3, neutral; 4, satisfied; 5, very satisfied. **b** Spectacle dependence score rated on a five-point scale as follows for near, intermediate, and distance vision: 1, never; 2, seldom; 3, about half the time; 4, usually; 5, always. An asterisk indicates *p* < 0.05 by the Student’s *t*-test

There was no significant difference in the incidence of glare, starburst, and halos between the ICB00 and ZCB00 groups (Table 5). The mean patient satisfaction score for near and intermediate vision was significantly higher in the ICB00 group (4.0 ± 0.9 and 4.4 ± 0.6, respectively) than in the ZCB00 group (3.2 ± 0.9 and 3.7 ± 0.8, respectively) (*P* = 0.026 and *P* = 0.017, respectively; Fig. 3). However, there was no significant difference in the mean patient satisfaction score for overall and distance vision between the two groups. The spectacle dependence score for near and intermediate vision was significantly better in the ICB00 group (1.7 ± 1.3 and 1.1 ± 0.3, respectively) compared to the ZCB00 group (3.2 ± 1.4 and 2.3 ± 1.4, respectively), but not that for distance vision (Fig. 3).

**Table 5:** Comparison of postoperative incidence of photic phenomena between the ICB00 and ZCB00 groups

	ICB00	ZCB00	*P* value*
Glare, n			
Yes: No (%)	2 (13.3): 13 (86.7)	3 (20.0): 12 (80.0)	> 0.999
Starbursts, n			
Yes: No (%)	0 (0.0): 15 (100.0)	1 (6.7): 14 (93.3)	> 0.999
Halos, n			
Yes: No (%)	1 (6.7): 14 (93.3)	0 (0.0): 15 (100.0)	> 0.999

*Fisher’s exact test

### Optical bench performance

**Fig. 4:**
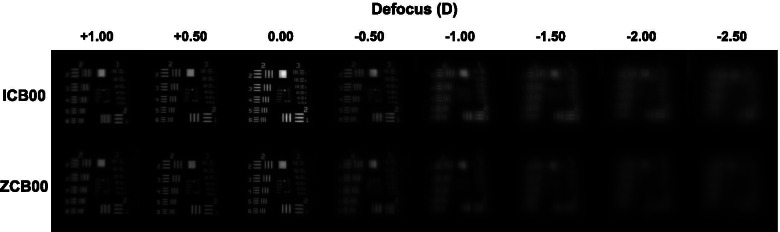
Captured images of the 1951 United States Air Force resolution test chart using the ICB00 and ZCB00 intraocular lenses. Minus diopter defocus represents near distance

Figure 4 shows captured images of the 1951 USAF resolution test chart from two IOLs. For the ZCB00 IOL, as the minus diopters were added, the image was gradually blurred, and the image became indistinguishable from − 1.00 D. However, for the ICB00 IOL, the image was identifiable until − 2.00 D was added.

**Fig. 5:**
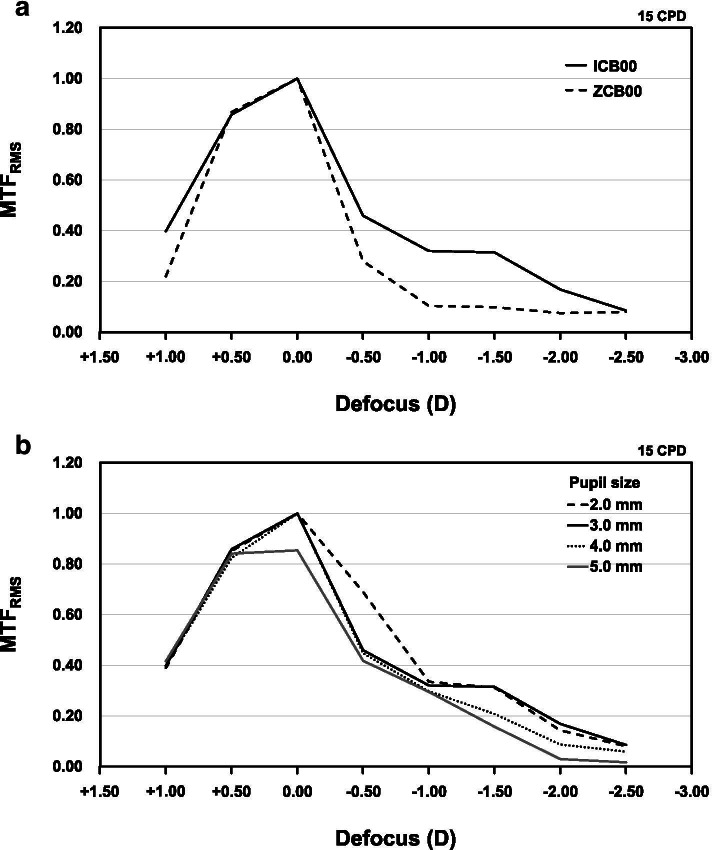
A comparison of the root mean square of modulation transfer function (MTF) values in the horizontal and vertical directions (MTF_RMS_). **a** A comparison of MTF_RMS_ values measured at a 3.0-mm pupil size between the ICB00 (solid line) and ZCB00 (dashed line) intraocular lenses. **b** A comparison of MTF_RMS_ values obtained with the ICB00 intraocular lens according to pupil size

MTF analysis showed that the ICB00 IOL had higher MTF_RMS_ values at − 0.50 D, − 1.00 D, − 1.50 D, and − 2.00 D of defocus (0.459, 0.320, 0.315, and 0.168, respectively) compared to the ZCB00 IOL (0.280, 0.104, 0.098, and 0.075, respectively) (Fig. 5A). This result is consistent with the defocus curves obtained in the clinical study.

The highest MTF_RMS_ values of the ICB00 IOL at − 0.50 D of defocus (0.690) was obtained in the pupil size of 2.0 mm (Fig. 5B and Table 6). On the other hand, the MTF_RMS_ values of the ICB00 IOL at between − 1.00 D to − 2.50 D of defocus was similar between the pupil size of 2.0 mm and 3.0 mm. This result indicates that best optical performance of the ICB00 IOL in the intermediate distance could obtained at 2.0 mm pupil size.

**Table 6:** A comparison of modulation transfer function (MTF) values in the horizontal and vertical directions and the root mean square of MTF values in those two directions obtained with the ICB00 intraocular lens according to pupil size

Defocus, D	Pupil size 2.0 mm			Pupil size 3.0 mm			Pupil size 4.0 mm			Pupil size 5.0 mm		
	MTF_Ver_	MTF_Hor_	MTF_RMS_	MTF_Ver_	MTF_Hor_	MTF_RMS_	MTF_Ver_	MTF_Hor_	MTF_RMS_	MTF_Ver_	MTF_Hor_	MTF_RMS_
1.00	0.348	0.427	0.389	0.134	0.547	0.398	0.241	0.509	0.398	0.276	0.520	0.416
0.50	0.867	0.837	0.852	0.688	1.000	0.858	0.599	1.000	0.824	0.645	1.000	0.841
0.00	1.000	1.000	1.000	1.000	1.000	1.000	1.000	1.000	1.000	0.964	0.728	0.854
−0.50	0.781	0.585	0.690	0.342	0.552	0.459	0.288	0.563	0.447	0.314	0.500	0.418
−1.00	0.348	0.321	0.335	0.379	0.247	0.320	0.222	0.358	0.298	0.216	0.357	0.295
−1.50	0.278	0.342	0.312	0.209	0.392	0.315	0.249	0.155	0.207	0.079	0.208	0.157
−2.00	0.141	0.145	0.143	0.094	0.219	0.168	0.123	0.000	0.087	0.042	0.000	0.030
−2.50	0.114	0.000	0.080	0.096	0.072	0.085	0.083	0.000	0.059	0.024	0.000	0.017

*D* Diopters, *Ver* Vertical, *Hor* Horizontal, *RMS* Root mean square

## Discussion

This study showed that after the Eyhance ICB00 IOLs were implanted bilaterally, patients could obtain better intermediate and near vision than the ZCB00 IOL while maintained the distant vision. Moreover, there was no difference in the incidence of photic phenomena between the ICB00 and ZCB00 IOL. In the optical bench test, the Eyhance ICB00 IOL showed an excellent MTF curve at intermediate distances, and it was affected by the pupil size.

Although cataract surgery with monofocal IOL implantation is still the most common option, there is a gap between postoperative results and patient’s expectations as it limits to improve only distant vision. At this point, many investigators have developed other IOL designs to improve near and intermediate vision, but those IOLs also have limitations as they accompany unwilling phenomena such as reduced contrast sensitivity, halos, and glares [18–20]. Besides, as the optical technologies differ, the subjects to apply those advanced IOLs are limited. Therefore, the Eyhance ICB00 IOL could be a good option as it shares the same geometry with the monofocal 1-piece IOL and provides an improved intermediate and near vision, and better spectacle independence as well, without accompanying other unwilling phenomena.

Previous studies reported the Eyhance ICB00 IOL’s effectiveness as it provided improved intermediate vision compared to the monofocal 1-piece IOL [10, 21, 22]. In those previous studies, the Eyhance ICB00 IOL yielded better UIVA, higher spectacle independence at intermediate distance, and similar optical quality compared to the classic monofocal IOL, but provided similar distance and near visual acuities [10, 21, 22]. The results of this study also showed that the Eyhance ICB00 IOL produced better intermediate VA and similar optical quality compared to the ZCB00 IOL. However, binocular UNVA and spectacle independence at near distance for the ICB00 group were better than those of the ZCB00 group in this study. The reason for the discrepant results between the previous studies and this study in terms of near vision improvement by the Eyhance ICB00 IOL compared to the ZCB00 IOL may be due to race and study design. The previous two studies indicating that the Eyhance ICB00 IOL provides better intermediate vision but not near vision were conducted in a prospective manner in Europe. On the other hand, this study was conducted in a retrospective manner in Asia. In line with this study, a previous study conducted in a retrospective manner in Asia showed that the Eyhance ICB00 IOL provided superior intermediate and near vision compared to the ZCB00 IOL [23]. Therefore, the effect of the Eyhance IOL for improving near vision needs to be confirmed through a large-scale study.

Optical bench tests in this study supported clinical outcomes. The MTF curves of two IOLs at distances showed similar good results. In contrast, the Eyhance ICB00 IOL showed better outcomes at intermediate distances, especially within the range up to − 1.50 D. Besides, when evaluating the effect of pupil size on the MTF curves of the Eyhance ICB00 IOL, it showed the best results with the 2.0 mm pupil size at intermediate distances. At the same time, it had a minimal impact at distances. The unique anterior surface lies on the center deviation of the Eyhance ICB00 IOL may explain those results [24]. Previous study showed that the through-focus MTF curve of the ICB00 shifted to a myopic defocus of − 0.50 D at a 2.0 mm pupil size and the maximum MTF value was obtained at − 0.5 D defocus [25]. However, in this study, the maximum MTF value was obtained at 0.0 D defocus without myopic shift of the MTF curve at a 2.0 mm pupil size. Considering the myopic shift of the through-focus MTF curve in the previous study, it is thought that a relatively lower MTF value (0.459) was measured at − 0.5 D defocus without myopic shift of the MTF curve in this study.

One limitation of the current study is that the sample size was relatively small, the follow-up period was relatively short, and the study design retrospectively reviewed the medical records. The other is that we did not evaluate whether patients’ pupil size had impacts on visual acuity.

## Conclusions

In conclusion, the TECNIS Eyhance ICB00 IOL may be a good option for both clinicians and patients. It provided better intermediate and near vision than the TECNIS 1-piece IOL while maintaining excellent distance vision without worsening the visual symptoms. Moreover, the results of the optical bench test, which showed that the Eyhance ICB00 IOL had higher MTF values between − 0.50 D and − 2.00 D of defocus compared to the ZCB00 IOL, supported these clinical outcomes.

## Data Availability

The datasets used and/or analysed during the current study are available from the corresponding author on reasonable request.

## Abbreviations

IOL: Intraocular lens
CDVA: Corrected distance visual acuity
ACD: Anterior chamber depth
AL: Axial length
UDVA: Uncorrected distance visual acuity
UNVA: Uncorrected near visual acuity
DCNVA: Distance corrected near visual acuity
UIVA: Uncorrected intermediate visual acuity
DCIVA: Distance corrected intermediate visual acuity
USAF: United States Air Force
CMOS: Complementary metal-oxide-semiconductor
ISO: International Organization for Standardization
CPD: Cycle per degree
MTF: Modulation transfer function
RMS: Root mean square

## References

[1] Bourne RR, Stevens GA, White RA, Smith JL, Flaxman SR, Price H, Jonas JB, Keeffe J, Leasher J, Naidoo K, Pesudovs K, Resnikoff S, Taylor HR (2013). Causes of vision loss worldwide, 1990-2010: a systematic analysis. Lancet Glob Health.

[2] Asbell PA, Dualan I, Mindel J, Brocks D, Ahmad M, Epstein S (2005). Age-related cataract. Lancet.

[3] Pedrotti E, Carones F, Aiello F, Mastropasqua R, Bruni E, Bonacci E, Talli P, Nucci C, Mariotti C, Marchini G (2018). Comparative analysis of visual outcomes with 4 intraocular lenses: monofocal, multifocal, and extended range of vision. J Cataract Refract Surg.

[4] Mendicute J, Kapp A, Levy P, Krommes G, Arias-Puente A, Tomalla M, Barraquer E, Rozot P, Bouchut P (2016). Evaluation of visual outcomes and patient satisfaction after implantation of a diffractive trifocal intraocular lens. J Cataract Refract Surg.

[5] Häring G, Dick HB, Krummenauer F, Weissmantel U, Kröncke W (2001). Subjective photic phenomena with refractive multifocal and monofocal intraocular lenses: results of a multicenter questionnaire. J Cataract Refract Surg.

[6] Kim JH, Eom Y, Park SY, Choi SY, Hwang HS, Kim JH, Song JS, Kim HM (2020). Rainbow halos occur less following implantation of extended range of vision one-piece intraocular lenses vs diffractive bifocal intraocular lenses. Int J Ophthalmol.

[7] MacRae S, Holladay JT, Glasser A, Calogero D, Hilmantel G, Masket S, Stark W, Tarver ME, Nguyen T, Eydelman M (2017). Special report: American Academy of Ophthalmology Task Force consensus statement for extended depth of focus intraocular lenses. Ophthalmology.

[8] Bellucci R, Cargnoni M, Bellucci C (2019). Clinical and aberrometric evaluation of a new extended depth-of-focus intraocular lens based on spherical aberration. J Cataract Refract Surg.

[9] Rocha KM (2017). Extended depth of focus IOLs: the next chapter in refractive technology?. J Refract Surg.

[10] Mencucci R, Cennamo M, Venturi D, Vignapiano R, Favuzza E (2020). Visual outcome, optical quality, and patient satisfaction with a new monofocal IOL, enhanced for intermediate vision: preliminary results. J Cataract Refract Surg.

[11] Kim M, Eom Y, Song JS, Kim HM (2018). Comparative evaluation of refractive outcomes after implantation of two types of intraocular lenses with different diopter intervals (0.25 diopter versus 0.50 diopter). BMC Ophthalmol.

[12] Kim JW, Eom Y, Chung HW, Song JS, Jeong JW, Park SK, Kim HM (2020). Factors for good near and distance visual outcomes of multifocal intraocular lens with inferior segmental near add. Graefes Arch Clin Exp Ophthalmol.

[13] Koo YH, Lee CS, Kim JS, Shin MC, Kim EC, Kim MS, Hwang HS (2020). Measuring defocus curves of monofocal, multifocal and extended depth-of-focus intraocular lenses using optical bench test. J Korean Ophthalmol Soc.

[14] Alba-Bueno F, Vega F, Millán MS (2011). Design of a test bench for intraocular lens optical characterization. Journal of physics: conference series; 2011.

[15] Eom Y, Yang SK, Yoon EG, Choi JN, Ryu D, Kim DW, Kim JH, Song JS, Kim SW, Kim HM (2020). Multizonal design multifocal intraocular lens-induced astigmatism according to orientation. J Refract Surg.

[16] Kim MJ, Zheleznyak L, Macrae S, Tchah H, Yoon G (2011). Objective evaluation of through-focus optical performance of presbyopia-correcting intraocular lenses using an optical bench system. J Cataract Refract Surg.

[17] Yoo YS, Whang WJ, Byun YS, Piao JJ, Kim DY, Joo CK, Yoon G (2018). Through-focus optical bench performance of extended depth-of-focus and bifocal intraocular lenses compared to a monofocal lens. J Refract Surg.

[18] Liu J, Dong Y, Wang Y (2019). Efficacy and safety of extended depth of focus intraocular lenses in cataract surgery: a systematic review and meta-analysis. BMC Ophthalmol.

[19] de Silva SR, Evans JR, Kirthi V, Ziaei M, Leyland M (2016). Multifocal versus monofocal intraocular lenses after cataract extraction. Cochrane Database Syst Rev.

[20] de Vries NE, Webers CA, Touwslager WR, Bauer NJ, de Brabander J, Berendschot TT, Nuijts RM (2011). Dissatisfaction after implantation of multifocal intraocular lenses. J Cataract Refract Surg.

[21] Auffarth GU, Gerl M, Tsai L, Janakiraman DP, Jackson B, Alarcon A, Dick HB (2021). Clinical evaluation of a new monofocal IOL with enhanced intermediate function in patients with cataract. J Cataract Refract Surg.

[22] Cinar E, Bolu H, Erbakan G, Yuce B, Aslan F, Fece M, Emre S (2021). Vision outcomes with a new monofocal IOL. Int Ophthalmol.

[23] Kang KH, Song MY, Kim KY, Hwang KY, Kwon YA, Koh K (2021). Visual performance and optical quality after implantation of a new generation monofocal intraocular lens. Korean J Ophthalmol.

[24] Tognetto D, Cecchini P, Giglio R, Turco G (2020). Surface profiles of new-generation IOLs with improved intermediate vision. J Cataract Refract Surg.

[25] Vega F, Millán MS, Gil MA, Garzón N (2020). Optical performance of a monofocal intraocular lens designed to extend depth of focus. J Refract Surg.

